# Middle Cerebral Artery Ischemic Stroke Occurring in a Woman With Metastatic Breast Cancer: A Case Report

**DOI:** 10.7759/cureus.99709

**Published:** 2025-12-20

**Authors:** Maan Jamjoom, Wesam Edrees, Noura Alshareef, Rahaf Alrugi, Meaad Alshehri, Yara Alkowaileet, Reem A Adas

**Affiliations:** 1 Department of Emergency Medicine, King Abdullah Medical City, Jeddah, SAU; 2 Department of Emergency Medicine, Ministry of National Guard Health Affairs (MNGHA), Jeddah, SAU; 3 College of Medicine, King Saud bin Abdulaziz University for Health Sciences, King Abdullah International Medical Research Center, Jeddah, SAU; 4 Department of Radiology, Ministry of National Guard Health Affairs (MNGHA), Jeddah, SAU

**Keywords:** breast cancer, ischemic, metastatic, middle cerebral artery ischemic stroke, middle cerebral artery (mca), stroke

## Abstract

Brain metastasis refers to the spread of cancer cells from a primary tumor to the brain. A rare but serious complication of brain metastasis is middle cerebral artery (MCA) occlusion. This report discusses a case involving a 42-year-old woman with metastatic breast cancer who presented to the emergency department with symptoms of weakness, headache, mouth deviation, and dysarthria that had lasted for two hours. Prior to this incident, she had been in her usual state of health. Upon physical examination, it was noted that the patient had left-sided hemiparesis. A brain CT scan with contrast showed no changes compared to her baseline imaging. However, a stroke protocol CT angiogram and brain perfusion scan indicated an acute infarction in the right MCA. To treat her condition, complete recanalization was achieved successfully through mechanical thrombectomy. Following the procedure, a post-mechanical thrombectomy protocol was established, and the patient was admitted to the ICU. To reduce the risk of stroke recurrence, aspirin monotherapy was initiated. This case highlights the importance of a multidisciplinary approach when managing ischemic stroke in patients with metastatic cancer.

## Introduction

The spread of cancer cells from a primary tumor to the brain is known as brain metastasis. Some examples of types of cancer that are associated with brain metastasis are lung, breast, and melanoma cancers. This progression can result in various neurological complications with numerous symptoms and signs. Brain metastasis presents a significant challenge in advanced breast cancer cases, with approximately 10% to 15% of patients with metastatic breast cancer developing brain metastases. Notably, metastatic brain tumors are more prevalent in HER2+ and triple-negative breast cancer types [1]. Among the various forms of brain metastases, the occlusion of the middle cerebral artery (MCA) is a rare yet devastating complication that can lead to profound neurological impairments, significantly affecting the quality of life and prognosis of patients.

MCA occlusion stands as a leading cause of ischemic stroke, responsible for around 90% of anterior circulation infarcts. Typically occurring in the main stem (M1) or its branches (M2), this condition manifests through contralateral hemiplegia, sensory deficits, and, depending on the affected hemisphere, aphasia or hemisensory neglect. Given the critical brain regions supplied by the MCA, its occlusion can result in substantial neurological deficits, underscoring the importance of timely diagnosis and intervention to enhance outcomes and reduce brain damage [2,3].

## Case presentation

The patient was a 42-year-old female, a known case of metastatic breast cancer to the brain, liver, and bone, who presented to the emergency department (ED) with left-sided weakness and sensory loss, right mouth deviation, dysarthria, and a worsening headache for two hours.

History of presenting illness

**Figure 1:**
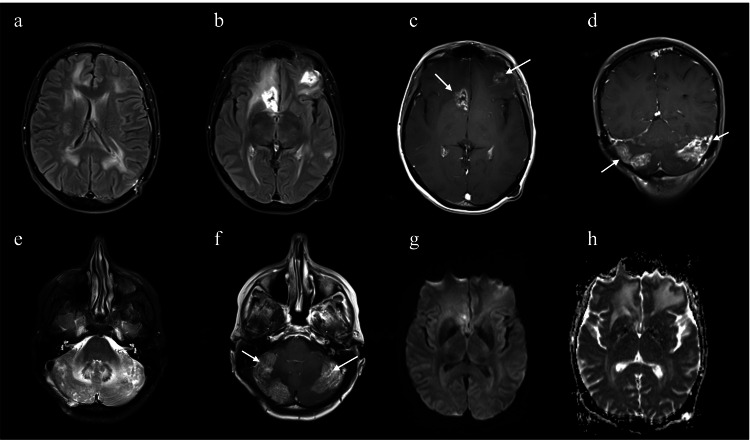
An MRI done two weeks before the stroke onset demonstrated the patient's known supra- and infratentorial metastatic infiltration, with enhancement (white arrows), edema, and mass effect. Diffusion-weighted imaging (DWI) (g, h) did not show any diffusion restriction to suggest an infarct.

Prior to the event, the patient was in her usual state of health with a baseline brain imaging, as per Figure 1.

The patient reported experiencing headaches accompanied by photophobia and phonophobia, without any visual disturbances. Additionally, baseline dysarthria was present, though speech remained understandable since the craniotomy two years ago. The patient also faced difficulty swallowing but denied any loss of consciousness, seizures, or injuries from a fall.

She was diagnosed with metastatic breast cancer in the brain, liver, and bone six years ago (invasive ductal carcinoma grade II, with lobular features, estrogen receptors 10%, progesterone receptor negative, HER2 +3). She has undergone multiple lines of systemic therapy, a craniotomy for tumor resection due to mass effect causing tonsillar herniation, and ventriculoperitoneal (VP) shunt placement two years ago. Currently, she is on palliative care and antidepressants.

Physical examination

The patient was conscious, alert, oriented, and appeared well but had been dysarthric since the craniotomy. Vital signs were normal, including serum glucose. Neurological examination showed equal and reactive pupils bilaterally with normal size. There was a left homonymous hemianopia (by visual threat), impaired pursuit, left upper motor neuron facial palsy, and moderate slurred speech. The left upper limb showed weakness (2/5) with hypotonia, and the left lower limb showed weakness (3/5) with normal tone. There is decreased pinprick sensation in the left upper and lower limbs. Plantar reflexes are upgoing bilaterally. The National Institutes of Health Stroke Scale (NIHSS) was 10. The right upper and lower limbs had full strength (5/5), intact sensation, and normal tone.

Laboratory investigation

The laboratory workup of the patient is presented in Tables 1, 2.

**Table 1:** Complete blood count reports F: female; M: male

Test	Result	Unit	Reference Range
Hemoglobin	9.3	g/dL	12–16 (F), 13.5–17.5 (M)
WBC	5.9	×10³/µL	4.0–10.0
Eosinophils %	0.0011	%	0–6%
Eosinophils #	0.187	×10³/µL	0.0–0.5
Neutrophils %	0.675	%	40–70%
RBC	3.6	×10⁶/µL	4.0–5.2 (F), 4.5–5.9 (M)
Hematocrit	0.29	%	36–46 (F), 40–52 (M)
Lymphocytes %	0.204	%	20–45%
Mean corpuscular volume	80.3	fL	80–100
Monocytes #	0.59	×10³/µL	0.2–1.0
Monocytes %	9.91	%	2–10%
Lymphocytes #	1.21	×10³/µL	1.0–3.0
Neutrophils #	4.01	×10³/µL	1.8–7.7
Mean corpuscular hemoglobin	25.8	pg	27–33
Mean corpuscular hemoglobin concentration	32.1	g/dL	32–36
Red cell distribution width	26.7	%	11.5–14.5
Platelets	488	×10³/µL	150–400
Nucleated red blood cells	0.02	×10³/µL	0
Mean platelet volume	8.51	fL	7–12

**Table 2:** Chemistry and liver profile with coagulation profile

Glucose (Point-of-Care (POC))			
Test	Result	Unit	Reference Range
Glu2 (POC)	5.8 mmol/L	mmol/L	4.0–7.8
Chemistry & Liver Profile			
Test	Result	Unit	Reference Range
Estimated glomerular filtration rate	101	mL/min/1.73m²	>90
CRP	<0.2	mg/dL	<0.5
Bilirubin (total)	7.4	µmol/L	3–21
Alkaline phosphatase	89	U/L	44–147
Total protein	65	g/L	60–80
Phosphate	1.23	mmol/L	0.8–1.5
Adjusted calcium	2.07	mmol/L	2.2–2.6
Potassium	4.4	mmol/L	3.5–5.0
Albumin	40	g/L	35–50
Gamma-glutamyl transferase	40	U/L	9–36
Magnesium	0.88	mmol/L	0.7–1.0
Sodium	139	mmol/L	135–145
Chloride	110	mmol/L	98–107
Coagulation profile			
Test	Result	Unit	Reference Range
International normalized ratio	1	—	0.9–1.1
Prothrombin time	12	sec	11–15
Partial thromboplastin time	23	sec	25–35

Imaging

**Figure 2:**
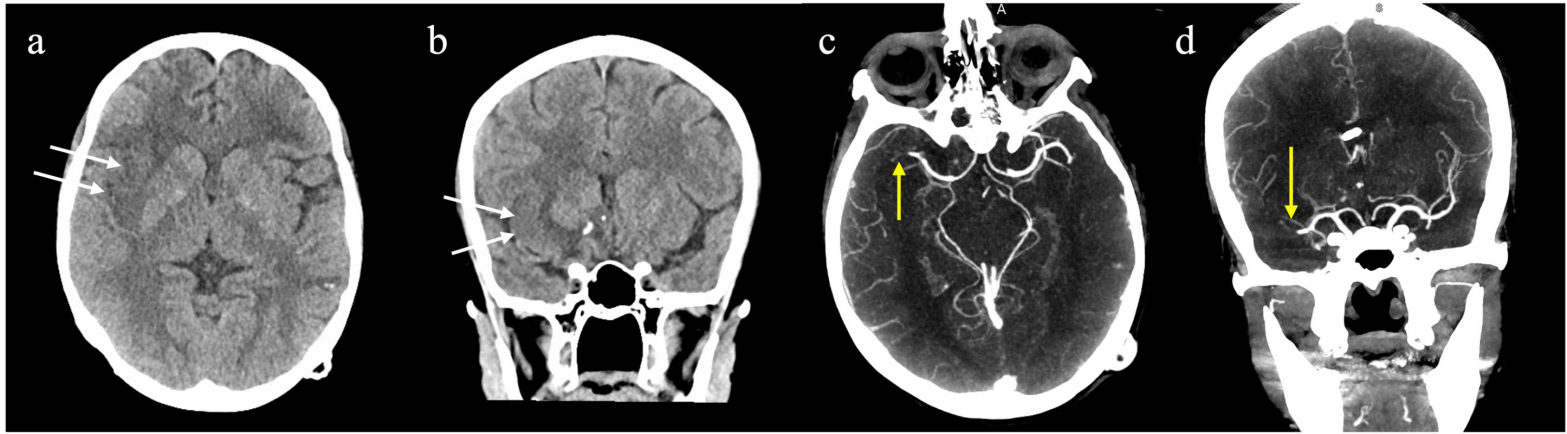
Stroke protocol CT imaging done at the time of presentation demonstrated findings of acute infarction with loss of insular ribbon sign (white arrows) on the unenhanced CT (a,b). The CT angiogram images (c,d) show the right middle cerebral artery (MCA) occlusion with abrupt cutoff of the enhancement (yellow arrows). The left MCA is patent.

**Figure 3:**
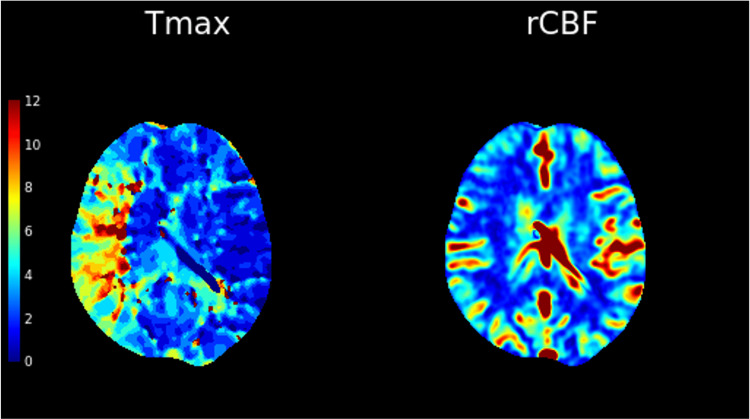
CT perfusion maps done as a part of the stroke protocol show a large area of perfusion mismatch between the time-to-maximum (Tmax) and the relative cerebral blood flow (rCBF) in the right middle cerebral artery territory. This signifies salvageable ischemic brain tissue that will benefit from endovascular treatment.

A brain CT scan with contrast was conducted in the ED. Reported no acute insult or new lesions. Stroke protocol CT imaging, CT angiogram, and perfusion scan of the brain uncovered a right MCA acute infarction and an abrupt contrast cutoff of the distal M1/proximal M2 segment of the right MCA, as per Figures 2, 3. This led to the decision that the patient was a candidate for urgent thrombectomy.

Plan

**Figure 4:**
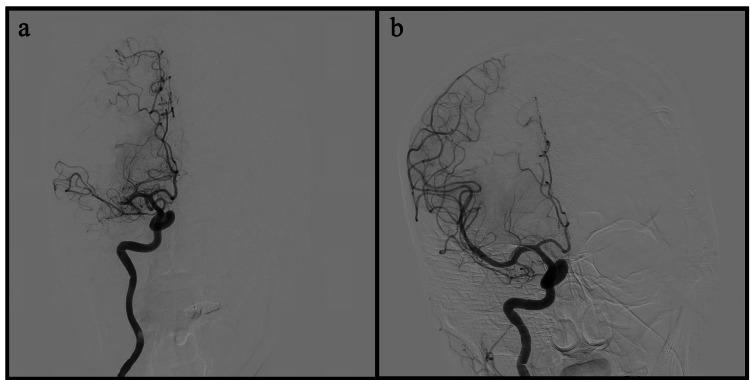
Frontal projections of a selective injection of the right internal carotid artery (ICA). The initial run (a) shows occlusion/cutoff of the right middle cerebral artery (MCA) with nonvisualization of the M3 or M4 branches. The post-thrombectomy injection (b) shows successful complete recanalization of the right MCA. Several variable-sized clots were removed (not shown).

The initial treatment phase involved the successful mechanical thrombectomy, which retrieved four clots resulting in complete recanalization (Figure 4). Following the procedure, the patient was admitted to the ICU and commenced on the post-mechanical thrombectomy protocol. Upon assessment after mechanical thrombectomy, the patient, although vitally stable, exhibited disorientation with a Glasgow Coma Scale score (GCS) of 14/15, left-sided hemiplegia, and hemisensory loss. Subsequent brain CT scans on the first and third days post-thrombectomy revealed expected changes in the right MCA territory acute infarction without acute hemorrhage. As a preventive measure, aspirin monotherapy was introduced to mitigate the risk of hemorrhagic transformation.

## Discussion

This case report describes the occurrence of ischemic stroke resulting from an acute MCA occlusion in a 42-year-old female patient with brain metastasis originating from breast cancer. The infrequency of this event presents a unique challenge in terms of clinical presentation and management. As aforementioned, the complexity of MCA occlusion in metastatic breast cancer underscores the need for specialized attention in addressing brain metastasis, particularly in advanced breast cancer cases with MCA involvement. The diagnostic process revealed a right MCA acute infarction and occlusion, emphasizing the intricate relationship between the patient's medical conditions and the development of neurological complications.

Cerebrovascular disease is a common occurrence in cancer patients, with around 15% experiencing thromboembolic events. Stroke patients with non-conventional mechanisms may be identified as having cancer-related strokes. Furthermore, a significant portion of cancer patients suffer strokes with non-conventional mechanisms, emphasizing the importance of recognizing and understanding stroke causes in this patient population [4].

Various theories have been proposed to explain the cause of stroke in cancer patients, but the exact mechanism remains unclear, including hypercoagulability, presence of an embolism, dyslipidemia, other comorbidities, and presence of risk factors such as smoking and chemotherapy [5]. Moreover, the intricate nature of ischemic strokes in the context of advanced cancer adds another layer of complexity to the management approach, necessitating a tailored care plan that addresses the interplay between cancer progression and cerebrovascular events.

The causes of stroke in cancer patients may vary from those in individuals without cancer. As a result, the treatment and preventive approaches for these two groups may differ [4]. The successful mechanical thrombectomy procedure performed in this case exemplifies the importance of prompt diagnosis and intervention in cases of MCA occlusion for optimizing patient outcomes and reducing neurological damage. A similar case reported by Takuya Moriyama et al. involving a 64-year-old male patient with lung cancer and tumor embolism leading to acute MCA occlusion also underwent mechanical thrombectomy. The patient showed rapid improvement in left-sided hemiparesis, left facial paresis, dysarthria, and left hemispatial neglect post procedure, with gradual disappearance of symptoms and restoration of ambulation, which emphasizes the efficacy of this intervention [6].

The integration of palliative care in managing patients with advanced breast cancer and concomitant ischemic stroke plays a pivotal role in addressing their different needs. The management of such complications requires a nuanced approach that considers both the oncological treatment strategies and the neurological interventions needed to address stroke-related deficits.

Collaborative efforts between oncologists, neurologists, and palliative care specialists are essential in providing holistic care that addresses the diverse needs of patients with these complex presentations. Palliative care not only focuses on symptom management and enhancing quality of life but also provides crucial support for patients and their families facing complex medical conditions.

## Conclusions

This case illustrates the infrequent occurrence of an acute MCA occlusion in a patient with metastatic breast cancer. Mechanical thrombectomy was successful in achieving full recanalization and avoiding further neurological deterioration.

Stroke in the setting of advanced malignant disease is usually due to cancer-related mechanisms and should be promptly recognized and targeted for treatment. There is yet no substitute for a multidisciplinary approach with careful attention given to oncologic, neurologic, and palliative care, to optimize outcomes and to enhance disease-specific quality of life for these patients.

## References

[1] (2025). Brain metastases: when cancer spreads to the brain. https://my.clevelandclinic.org/health/diseases/17225-metastatic-brain-tumors.

[2] Jichici D, Sharma AV. (2025). Anterior circulation stroke. https://emedicine.medscape.com/article/1159900-overview.

[3] (2025). Middle cerebral artery occlusion. https://www.sciencedirect.com/topics/medicine-and-dentistry/middle-cerebral-artery-occlusion.

[4] Kim SG, Hong JM, Kim HY (2010). Ischemic stroke in cancer patients with and without conventional mechanisms: a multicenter study in Korea. Stroke.

[5] Karkra R, Jain R, Shivaswamy RP (2023). Recurrent strokes in a patient with metastatic lung cancer. Cureus.

[6] Moriyama T, Sugiura Y, Hayashi Y (2021). Mechanical thrombectomy for acute middle cerebral artery occlusion caused by tumor embolism: a case report. J Neuroendovasc Ther.

